# Rapid rehabilitation nursing improves clinical outcomes in postoperative patients with colorectal carcinoma: A protocol for randomized controlled trial

**DOI:** 10.1097/MD.0000000000022857

**Published:** 2020-11-06

**Authors:** Genying Zhu, Chen Wu, Xiaoying Shen

**Affiliations:** Department of Gastrointestinal Surgery, Huzhou Central Hospital (Affiliated Central Hospital of HuZhou University), Zhejiang, China.

**Keywords:** colorectal carcinoma, fast track surgery, nursing, protocol

## Abstract

**Background:**

Colorectal carcinoma has a high incidence rate and the high mortality rate has always been an important global health challenge. Surgical treatment is widely performed in patients with colorectal carcinoma. Fast track surgery (FTS) applies evidence-based medical concept to optimize the management during the operation, so as to reduce the psychological and physical trauma stress of surgical patients and make them recover rapidly. We perform this protocol for randomized controlled study to evaluate the efficacy of a rapid rehabilitation care in colorectal carcinoma surgery.

**Methods:**

It is a single-center randomized controlled study to be conducted from January 2021 to December 2021. It was authorized via the Ethics Committee of the Huzhou Central Hospital (20191127-01). Eighty participants who undergo colorectal carcinoma surgery will be included in this research. Patients are randomly assigned to control group (standard management group, including 40 samples) and study group (the FTS group, including 40 samples). The main results are times of postoperative exhaust, first defecation, ambulation, first eating, and postoperative hospital stay. Secondary outcomes are incidence of nausea and emesis, wound infection, urinary tract infection, lung infection, deep vein thrombosis, and rehospitalization rate among the 2 groups. All analyses are conducted using the SPSS for Windows Release 15.0.

**Results:**

Figure 1 shows the clinical results between groups.

**Conclusion:**

The research can offer a reliable basis for the effectiveness of a rapid recovery nursing program in patients with colorectal carcinoma.

**Trial registration:**

This study protocol was registered in Research Registry (researchregistry6038)

## 1 Introduction

Colorectal carcinoma has a high incidence rate and the high mortality rate has always been an important global health challenge.^[1,2]^ About 10% of deaths occur annually in colorectal carcinoma, and the incidence rate and mortality rate of men is one-fourth higher than that of women.^[3]^ The incidence rate varies in different geographical locations, and is often higher in most developed countries. With the rapid progress of developing countries, it is estimated that there will be 2.5 million colorectal carcinoma patients in the world by 2035.^[4]^ However, in highly developed countries, the incidence of colorectal carcinoma is becoming stable and decreasing. This is mainly due to the increase of colorectal carcinoma related screening programs and colonoscopy in developed countries, as well as lifestyle and diet management.^[5]^ Although genetic factors, obesity, lifestyle, and living environment are associated with the disease, the specific relationship is not yet fully clear.

Surgical treatment is widely performed in patients with colorectal carcinoma.^[6]^ However, several patients are associated with several postoperative complications, which delays recovery. Fast track surgery (FTS) applies evidence-based medical concept to optimize the management during the operation, so as to reduce the psychological and physical trauma stress of surgical patients and make them recover rapidly.^[7,8]^ Auxiliary nursing is an important procedure in the treatment of carcinoma, which is used to meet the physical and mental needs, social needs, information acquisition, and actual needs of patients.^[9]^ The concept of continuous care during surgery has been successfully accepted in surgery.^[10]^ Li et al^[11]^ reported that FTS nursing program can efficaciously relieve the bad mood and pain of patients with hip fracture. Currently, few studies have investigated the clinical effect of FTS in perioperative nursing of colorectal carcinoma surgery. We perform this protocol for randomized controlled study to evaluate the efficacy of a rapid rehabilitation care in colorectal carcinoma surgery.

## 2 Materials and methods

### 2.1 Study design

It is a single-center randomized controlled study to be conducted from January 2021 to December 2021. This study is performed according to the SPIRIT Checklist of randomized researches. It was admitted via the Ethics Committee of the Huzhou Central Hospital (20191127-01), and it has been registered in the research registry (researchregistry 6038).

### 2.2 Inclusion and exclusion criteria

Inclusion criteria included colorectal carcinoma testified by pathology; and patients willing to participate in the survey and finish the chart independently. Exclusion criteria included patients with psychiatric history; patients taking psychotropic drugs within the last 6 months; patients with palliative gastrectomy; and patients with other severe visceral diseases.

### 2.3 Subjects

Eighty participants who undergo colorectal carcinoma surgery will be included in this research. In the random envelope, a random number is assigned to whole patients through the random-number table, and the distribution result is invisible. Patients are assigned randomly to control group (standard management group, including 40 samples) and study group (the FTS group, including 40 samples).

### 2.4 Intervention

#### 2.4.1 Study group

Preoperative preparation. The medical personnel explains common sense in nursing, first-aid steps, and how to act in concert with medical personnel during perioperative period. Before surgery, the staff builds a trusting relationship with patients to enhance their obedience, and provides psychological consulting to help patients deal with preoperative anxiety and fear of surgery.

Intraoperative steps. Short effect general anesthesia and short effect regional block anesthesia are used in the operation. During the operation, the fluid should be strictly regulated, and the patient's normal temperature should be maintained with a heater to prevent heart overload, water intoxication, cell swelling, and other complications.

Postoperative care. Nonsteroidal anti-inflammatory drugs (NSAIDs) and self-control analgesia are given after operation. The first day after operation, the patients are in bed for 4 hours. Low power red laser is applied to irradiate the surgical area to promote the wound healing. Antibiotics are used on the first day after operation, and the bladder balloon is dislodged the following day.

#### 2.4.2 Control group

Preoperative preparation. Patients take cathartic orally and receive clean enema.

Intraoperative steps. A nasogastric tube is inserted until the passage of gas by anus, and general anesthetics are during the operation.

Postoperative care. After surgery, dolantin, morphine, and other opioid agents are used for analgesia. Before normal eating, intravenous injection of nutrient solution is used as nutritional supplement, and antibiotics are stopped after the blood picture and body temperature return to normal level.

### 2.5 Outcomes

The main results are times of postoperative exhaust, first defecation, ambulation, first eating, and postoperative hospital stay. Secondary outcomes are incidence of nausea and emesis, wound infection, urinary tract infection, lung infection, deep vein thrombosis, and readmission rate between the 2 groups.

### 2.6 Statistical analysis

All analyses are conducted using the SPSS for Windows Release 15.0. All results are expressed with appropriate form, for instance, mean, median, standard deviation, and percentage. The comparison between the 2 groups is conducted through applying the independent samples *t* test or the Mann–Whitney *U* test. And for the contrast of categorical variables between the groups, it can be implemented with Chi-square test. The *P* < .05 means that the results are statistically significant.

## 3 Results

**Figure 1:**
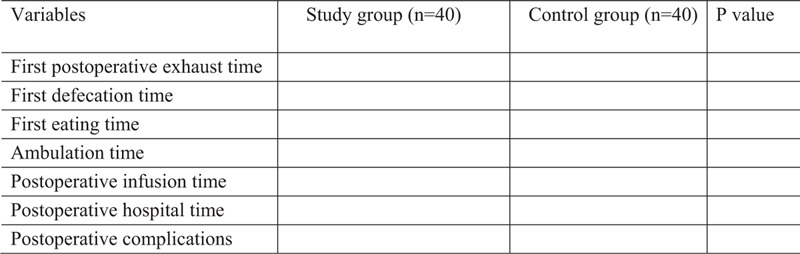
The clinical results between groups.

Figure 1 shows the clinical results between groups.

## 4 Discussion

As a new surgical nursing pattern, FTS is broadly adopted in gynecology, orthopedics, and general surgery.^[12–14]^ It refers to the cooperation of nurses, anesthesiologists, specialists, patients’ families, nutritionists, and rehabilitation therapists to minimize the stress reaction during the operation, promote the postoperative recovery, and lower the hospitalization time and costs.^[15]^ Its concept is quite different from the former concept, which greatly impacts and changes the existing surgical treatment methods.^[16]^ For colorectal carcinoma surgery, the conventional concept emphasizes the need of routine preoperative bowel preparation, such as enema cleaning.^[17]^ Recent studies have found that excessive bowel preparation can cause intestinal flora imbalance and injure the intestinal natural barrier, thus increasing the probability of postoperative abdominal infection. At the same time, long-term fasting of water and food will cause the dehydration, resulting in water and electrolyte balance disorder, which is not beneficial to postoperative recovery and improvement of nutritional status. This is the first randomized controlled study to assess the efficacy of a rapid rehabilitation care in colorectal carcinoma surgery. However, the sample size we used is relatively small. In order to confirm the results, the larger investigations are required.

## 5 Conclusion

The research can offer a reliable basis for the effectiveness of a rapid recovery nursing program in patients with colorectal carcinoma.

## Author contributions

Xiaoying Shen plans the study design. Chen Wu reviews the protocol. Chen Wu collects data. Genying Zhu writes the manuscript. All authors approve the submission.

**Conceptualization:** Chen Wu.

**Data curation:** Chen Wu.

**Formal analysis:** Chen Wu.

**Funding acquisition:** Xiaoying Shen.

**Writing – original draft:** Genying Zhu.

**Writing – review & editing:** Genying Zhu.
